# Fuzzy association rule mining and classification for the prediction of malaria in South Korea

**DOI:** 10.1186/s12911-015-0170-6

**Published:** 2015-06-18

**Authors:** Anna L. Buczak, Benjamin Baugher, Erhan Guven, Liane C. Ramac-Thomas, Yevgeniy Elbert, Steven M. Babin, Sheri H. Lewis

**Affiliations:** Johns Hopkins University Applied Physics Laboratory, 11100 Johns Hopkins Rd, Laurel, MD 20723-6099 USA

**Keywords:** Malaria, Prediction, Association rule mining, Fuzzy logic, Classification, Environmental data, Socio-economic data, Epidemiological data

## Abstract

**Background:**

Malaria is the world’s most prevalent vector-borne disease. Accurate prediction of malaria outbreaks may lead to public health interventions that mitigate disease morbidity and mortality.

**Methods:**

We describe an application of a method for creating prediction models utilizing Fuzzy Association Rule Mining to extract relationships between epidemiological, meteorological, climatic, and socio-economic data from Korea. These relationships are in the form of rules, from which the best set of rules is automatically chosen and forms a classifier. Two classifiers have been built and their results fused to become a malaria prediction model. Future malaria cases are predicted as ***LOW***, ***MEDIUM*** or ***HIGH***, where these classes are defined as a total of 0–2, 3–16, and above 17 cases, respectively, for a region in South Korea during a two-week period. Based on user recommendations, ***HIGH*** is considered an outbreak.

**Results:**

Model accuracy is described by Positive Predictive Value (PPV), Sensitivity, and F-score for each class, computed on test data not previously used to develop the model. For predictions made 7–8 weeks in advance, model PPV and Sensitivity are 0.842 and 0.681, respectively, for the ***HIGH*** classes. The F0.5 and F3 scores (which combine PPV and Sensitivity) are 0.804 and 0.694, respectively, for the ***HIGH*** classes. The overall FARM results (as measured by F-scores) are significantly better than those obtained by Decision Tree, Random Forest, Support Vector Machine, and Holt-Winters methods for the ***HIGH*** class. For the ***MEDIUM*** class, Random Forest and FARM obtain comparable results, with FARM being better at F0.5, and Random Forest obtaining a higher F3.

**Conclusions:**

A previously described method for creating disease prediction models has been modified and extended to build models for predicting malaria. In addition, some new input variables were used, including indicators of intervention measures. The South Korea malaria prediction models predict ***LOW***, ***MEDIUM*** or ***HIGH*** cases 7–8 weeks in the future. This paper demonstrates that our data driven approach can be used for the prediction of different diseases.

## Background

Malaria is a parasitic disease of humans and is transmitted via the bite of infectious female *Anopheles* mosquitoes. The adult female mosquito requires a blood meal in order to obtain the proteins necessary to complete the development of her eggs [1]. The salivary glands of infectious mosquitoes carry parasites belonging to the genus *Plasmodium*, with *P. vivax* and *P. falciparum* being the most common pathogens for humans. Uninfected mosquitoes acquire the parasite when they feed on infected hosts, thereby completing the transmission cycle. The time between parasite ingestion by the mosquito and the mosquito becoming infectious is known as the extrinsic incubation period (typically 10–21 days but varies with species and temperature). If the infectious mosquito doesn’t survive longer than this, then malaria won’t be transmitted. These mosquitoes often breed in stagnant water commonly found in ditches, rice fields, and impounded waters associated with human populations [2, 3]. The *Anopheles* mosquito has a flight range of 10 km or more [4, 5]. In order for malaria to spread among humans living in a region, the mosquitoes must be present so that the parasite can complete its life cycle (although there have been cases of person-to-person transmission via placenta, transfusion, organ transplantation, and shared needles) [6]. Human infection has an intrinsic incubation period, defined as the time between the mosquito bite introducing the parasite into the blood stream and the appearance of symptoms, varying from 7 to 30 days. The incubation period for *P. falciparum* tends to be at the shorter end of this time range. The symptoms of malaria can range from minimal to severe to death. Classically, malaria is characterized by high periodic fevers separated by phases of relatively normal body temperature.

Malaria has been reported to have a greater impact on human history than other infectious diseases and was found as far north as Canada before the American Civil War [6]. Globally, there were an estimated 207 million malaria cases and an estimated 627, 000 malaria deaths in 2012 [7]. Most malaria deaths globally occur in children under 5 years of age. Malaria caused by *P. falciparum* has become more drug resistant over recent years [8]. Also, increasing resistance of *Anopheles* mosquitoes to insecticides may impact the efficacy of malaria control [9]. Concerns have been expressed about the potential for changes in global malaria distribution due to the effects of climate change [1, 2, 6]. As an illustration of the continuing potential for the spread of this disease long after it was eradicated, *P. vivax* was isolated from local mosquitoes after human cases were identified in northern Virginia in the United States in 1998 and 2002 [10]. Changes in rainfall patterns during 1990–2009 may have contributed to the re-emergence of malaria in the northern Anhui Province of China [11]. After indigenous transmission disappeared in the 1970s, malaria caused by *P. vivax* re-emerged in the Republic of Korea (ROK, or South Korea) in 1993 and subsequent annual numbers of cases began to increase geometrically [12, 13]. Predicting malaria cases in ROK is the focus of this paper and is the result of discussions with public health professionals in ROK.

Studies of disease prediction differ in exactly what they predict, which may include transmission stability indices, vector abundance (e.g., ecological niche models), transmission suitability for a region (e.g., [14]), and human malaria incidence or prevalence. Malaria risk maps (e.g., [15]) are also useful, as they represent the outcomes of disease transmission models based on spatial and temporal data [16]. Corley et al. [17] noted that the difference between risk assessment and event prediction is that the former provides the risk of an outbreak occurring under specified conditions, while the latter provides the location and time period during which a disease outbreak will occur.

Environmental data are often used in prediction models as a proxy for vector abundance and vector-human interaction because these data are less labor intensive and expensive to collect than those from field studies. Historical malaria incidence rates can be used to indicate the presence of the parasite in the local human population. Therefore, some predictive studies used both environmental data and historical incidence data. For example, Abeku et al. [18] developed a linear mixed model to predict monthly human incidence of *P. falciparum* malaria for the present month (t = 0) using monthly malaria incidence at month t-1, monthly rainfall at t-2 and t-3, and average minimum monthly temperature at t-1. This model thus required all the previous months’ (t-1, etc.) data immediately prior to the month being predicted. Such rapid data availability (e.g., having the May monthly incidence data available on May 31 or June 1 in order to run the model) is rarely achieved in practice. Also, as noted by Zinszer et al. [19], these types of models do not naturally account for serial autocorrelation and failure to do so may bias the estimation of predictor effects and underestimate standard errors. While these types of models may show high correlation looking retrospectively, this does not necessarily indicate future performance with data not used in model development.

Corley et al. [17] systematically reviewed disease prediction models published in articles, books, theses, proceedings, and technical reports through December 2010. Their search was focused on models defined as “abstract computational, algorithmic, statistical, or mathematical representation that produces informative output related to event detection or event risk.” The reviewed models required a priori knowledge, and included those that were used to detect or predict an event, assess risk, or used to understand the drivers and dynamics of the event. From over twelve thousand citations, they found 44 papers that met their model criteria. These were classified as risk assessment models, event prediction models, spatial models, dynamical models, and event detection models. Corley et al. [17] found only four event prediction models (the type described in this article) and suggested that this was because of the difficulty in creating a model that truly predicts disease events.

Zinszer et al. [19] published a scoping review of models that specifically forecasted malaria incidence, prevalence, or epidemics. Their reviewed models had to include prior malaria incidence, prevalence, or epidemics as a predictor. The studies were further restricted to autochthonous transmission among human populations. They identified 29 studies that met their inclusion criteria but did not assess them for quality. They noted that prediction accuracy could not be compared because no common scale-independent measures were used in any of the reviewed studies. There were studies that used all available data in model development, without using a separate set for assessing prediction accuracy. They concluded that prediction accuracy should always be measured on reserved data (i.e., data not used in model development) and common prediction measures should be used to allow model comparison. In the review by Corley et al. [17], only six out of forty-four disease prediction model papers used reserved data for testing the models. An example of a model that used reserved data for testing is found in Briet et al. [20]. Like Zinszer et al. [19], Corley et al. [17] emphasized the importance of testing on reserved data to avoid bias.

The work presented here represents further development of the techniques of Buczak et al. [21, 22] for data mining disparate sources of data to create models for disease prediction. The technique for creating the malaria model was similar to that for creating the dengue models, but the resulting malaria model was distinct. In addition, the predictor variables for malaria were taken from the literature, so overall there is a different set of predictor variables for malaria than for dengue. The data mining approach avoids the assumption that a simple correlation analysis of past data will remain accurate for future data. Conservative estimates of model prediction accuracy are provided by testing the model on data not used in its development, so that potentially biased accuracy measures are avoided. In order to be more realistic operationally, the model does not rely on data that may not be available on the date the prediction is made. Finally, the model is able to take into account complex temporal and spatial relationships among the variables that would be missed by traditional correlation analyses. While the previous studies involved dengue fever in Peru [21] and The Philippines [22], this study uses a similar technique to create new models that focus on malaria in regions of the ROK near the border with the Democratic People’s Republic of Korea (DPRK, or North Korea), where most ROK malaria cases occur [13].

## Methods

### Predictor variables

As described in Buczak et al. [21, 22], the first step involves a review of the literature to find variables associated with the disease of interest, which in this case is malaria. Variables used for input into the prediction model include epidemiological, environmental, and socioeconomic data found to be useful in previous studies. Zinszer et al. [19] noted the importance of including transmission-reducing interventions to improve malaria outbreak prediction. Linthicum et al. [13] noted that malaria outbreaks in ROK were possibly a result of infected mosquitoes entering from DPRK. As mentioned earlier, the flight range of Anopheles mosquitoes has been estimated at 10 km or more [4, 5]. Therefore, we have also included DPRK mosquito net data, external funding for mosquito control sent to DPRK, and yearly malaria data for DPRK as predictor variables. Socioeconomic variables include population density in addition to the DPRK funding data.

Originally, the variables have different spatiotemporal scales, but all the variables need to fit the same spatiotemporal scale for the prediction model to work. The spatiotemporal scale was selected based on the distribution of the ROK malaria case data and is described next in more detail. For spatial variables the Geographic Information System (GIS) shape of each location is used to calculate values on a grid which is bounded by that location shape. The resolution of the grid is based on the original source of the data set. In this work the data sets have resolutions of 0.25, 0.05 and 1/120°. The values from each grid element that overlaps to the location shape are then used to obtain a single mean value for that location. For temporal variables, data were converted to weekly values, as described below. For a more detailed discussion on how these different types of data were processed and converted to the same spatiotemporal scale, the reader is referred to Buczak et al. [21, 22].

#### ROK malaria case data

Malaria weekly data were obtained from the Korea Centers for Disease Control and Prevention website (http://is.cdc.go.kr/dstat/index.jsp) and were interpreted in consultation with public health users in ROK. The smallest temporal resolution available was weekly. Therefore, all other input variables were converted to weekly intervals. In order to have a consistent definition of a week, weeks are numbered using the US Centers for Disease Control and Prevention (CDC) convention [23], with weekly intervals beginning on Sundays. ROK is divided geographically into 16 regions, of which there are 5 types: provinces, special autonomous provinces, special cities, metropolitan cities, and special autonomous cities. Most malaria cases in ROK occur in regions near the demilitarized zone (DMZ) that forms the border with the DPRK [13]. Therefore, case data were obtained for the following political divisions: Seoul (special city), Gyeonggi (province), Gangwon (province), and Incheon (metropolitan city). These political divisions are further subdivided, ranging from larger to smaller, into *gun* (county), *gu* (district), and *si* (city). For example, the special city of Seoul is divided into 25 gus, while the province of Gangwon is divided into 7 sis and 11 guns.

**Fig. 1:**
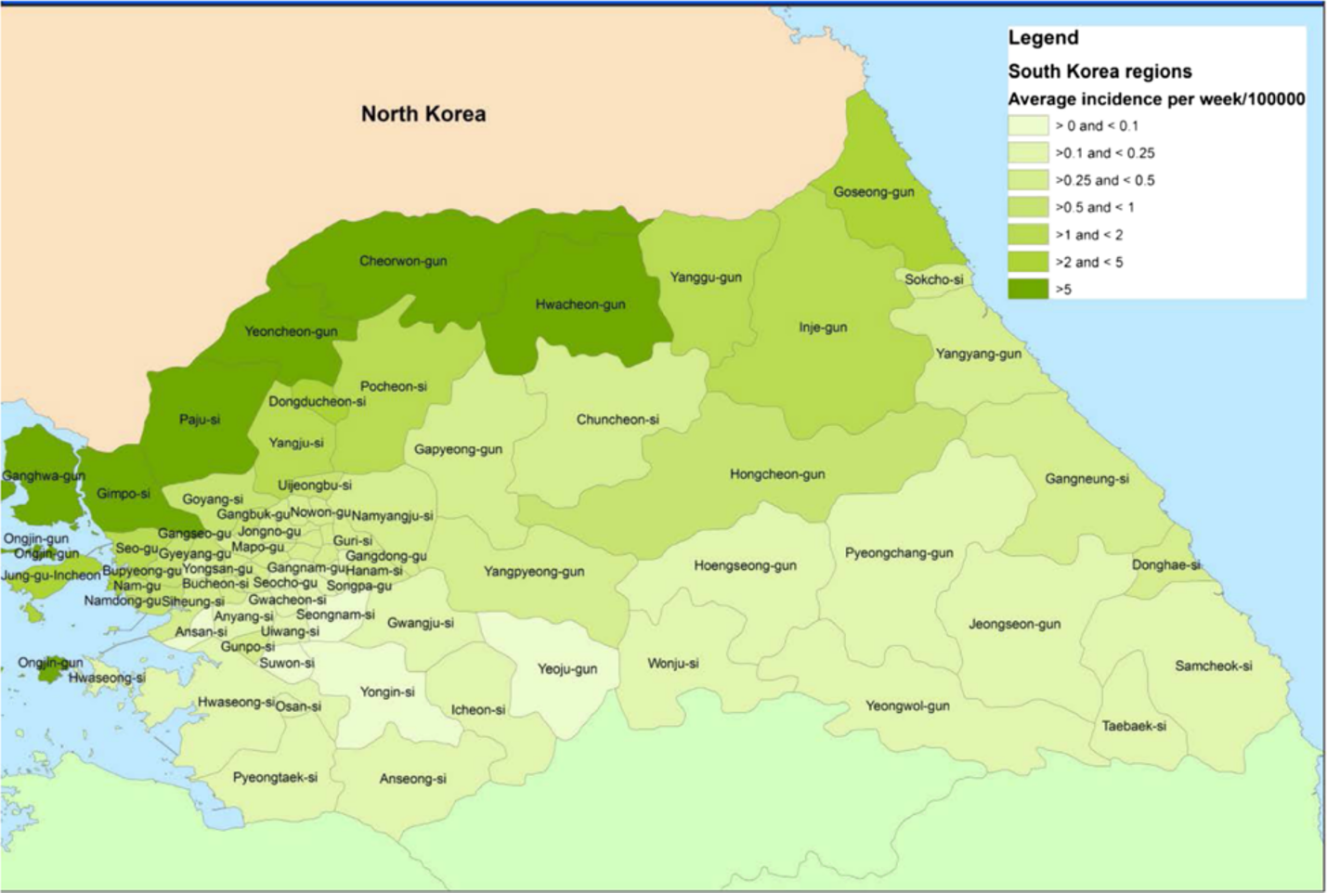
Average weekly incidence of malaria per 100,000 population in South Korea

**Fig. 2:**
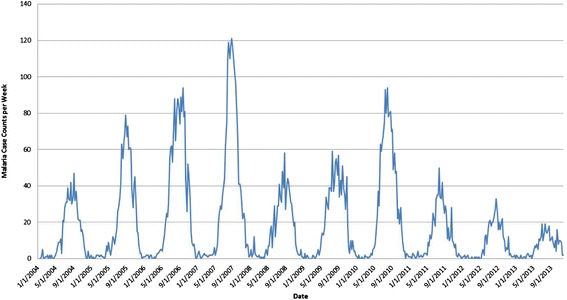
Combined malaria case counts for four northern provinces of South Korea

For the purpose of the analysis, the malaria case data for each of the political divisions and for each of their subdivisions (hence called regions) were extracted for the period from 2004–2013. To illustrate the distribution of malaria in the areas of interest, Fig. 1 provides a map of average weekly malaria incidence per 100,000 people for the entire period for each region. Figure 2 shows the combined malaria case counts for the four northern provinces of ROK. Consultation with ROK public health professionals determined that they were interested in a model that predicted malaria cases up to 8 weeks in advance for these specific provinces of ROK.

#### DPRK malaria case data

DPRK has reduced malaria within its borders by 90 % since 2001, from more than 140,000 to fewer than 15,000 annually. 70 % of DPRK cases of malaria are concentrated in the southern provinces bordering the DMZ [24]. Transmission of malaria in the DPRK is unstable, with a high potential for outbreaks during the June-September transmission season [12]. The yearly DPRK malaria case data come from the World Malaria Report 2013 [7], where Annex 6c lists yearly reported malaria cases for the whole DPRK. These DPRK malaria case data were included as one of the predictor variables.

#### DPRK mosquito net data

One potentially valuable explanatory variable for the load of malaria cases in a given year is the amount of malaria control measures provided to and administered by the DPRK. The most reliable source of malaria-prevention measures for DPRK is the world malaria report published by World Health Organization (WHO) [7]. One of the most consistently reported measures is the number of insecticide-treated nets that were sent to DPRK as part of their malaria prevention campaign. These numbers from WHO reports were tabulated for years 2008–2013 and are used as inputs to the prediction system.

Additional information was provided by the Malaria Control Project in the DPRK, sponsored by the Korean Sharing Movement and Provincial Government of Gyeonggi of ROK. From 2008 to 2011, a number of prevention measures including malaria nets were delivered to several DPRK provinces. Unfortunately the project was stopped in DPRK in 2011. The available numbers are used as inputs to the prediction system.

#### Financial data for DPRK malaria control

The WHO World Malaria Report [7] – Annex 3 “Funding for Malaria Control” describes the financial aid that each country receives for malaria control. In the case of DPRK, the main source of aid is the UNICEF Global Fund project [25], in addition to some aid from WHO and funds spent by the DPRK government. These funding data were used in developing the malaria case prediction.

#### Distance of ROK locations from the DMZ

It was mentioned earlier that *Anopheles* mosquitoes have been reported to travel 10 km or more [4, 5]. Because the mosquito vector may originate from the area north of and adjacent to the DMZ [12, 13], a potential explanatory variable representing the distance of each ROK region to the DMZ in kilometers was studied. Once the location shape files were entered into the database, a Structured Query Language (SQL) query was run to calculate the minimum distances from each region to the DMZ. The query was based on GIS database functions and returned the distance between the closest points of one shape to the other. The DMZ GIS shape encompasses the entire DMZ at the border between ROK and DPRK.

#### Elevation

Elevation data with 1/120° resolution were obtained from the NOAA National Geophysical Data Center website [26]. Any missing data (typically for ocean locations) were assigned an elevation of zero. The shape of the region overlapped to a 1/120° resolution grid is used to compute a single mean value of elevation for that region.

#### Rainfall

Rainfall data with 0.25° resolution were obtained from the NASA Tropical Rainfall Measuring Mission (TRMM) website [27], which contains satellite data measured by the TRMM instrument. These data contained hourly rainfall rates averaged over three-hour intervals. To convert from rainfall rates to rainfall amounts, all data were multiplied by three (the number of hours in the measurement interval) and the resulting data were aggregated into daily and then weekly rainfall totals. The shape of the region is overlapped to a 0.25° resolution grid and used to compute a single cumulative value of rainfall for that region. The rainfall data were then converted to weekly cumulative values for each ROK region for which the predictions are made. In addition, weekly cumulative values of the rainfall for the southern part of DPRK were calculated.

#### Land surface temperature

Using the Moderate Resolution Imaging Spectrometer (MODIS) instrument, satellite measurements are made of daily daytime and nighttime temperatures with 0.05° resolution. These data were obtained from the United States Geological Survey (USGS) Land Processes Distributed Active Archive Center using their website [28]. The shape of the region overlapped to a 0.05-resolution grid is used to compute a single mean value of day temperature and a single mean value of night temperature for each region. Day and night temperature data were each converted to weekly mean values for each ROK region for which the predictions are made. Weekly mean values of the daytime and nighttime temperatures for the southern part of DPRK near the DMZ were also calculated.

#### Vegetation indices: NDVI and EVI

Satellite MODIS measurements contain leaf area indices, including the Normalized Difference Vegetation Index (NDVI) and the Enhanced Vegetation Index (EVI). NDVI seasonal variations closely follow human-induced patterns, such as landscape disturbance [29]. Zinszer et al. [19] noted that human land use patterns are important but not often considered in malaria prediction models. EVI is calculated similarly to NDVI, but is considered to be more responsive than NDVI to canopy structural variations. Together, NDVI and EVI provide a surrogate assessment of green leaf biomass, photosynthetic activity, and the effects of seasonal rainfall, which are indicators of vector habitat characteristics. We obtained 16-day interval Normalized Difference Vegetation Index (NDVI) values and Enhanced Vegetation Index (EVI) values with 0.05° resolution from the USGS Land Processes Distributed Active Archive Center [28]. NDVI and EVI data were downloaded for ROK and DPRK. The shape of each region overlapped to a 0.05° grid is used to compute one mean value of NDVI and of EVI for each ROK region for a given time period. Similarly, mean values of the vegetation indexes for the southern part of DPRK are obtained. 16-day mean values were processed to obtain single-week averages to be coincident with weekly malaria case data, as described in Buczak et al. [21].

#### Southern oscillation index

The Southern Oscillation Index (SOI) is used to indicate the presence of an El Nino or La Nina climate anomaly period and the relative strength of that anomaly. SOI is based on the pressure difference between Darwin (Australia) and Tahiti (French Polynesia), which influences the strength of the prevailing easterly winds. The SOI data provide a measure of the El Nino Southern Oscillation (ENSO) climate effect, which can impact the weather in ROK [13]. Monthly SOI values were obtained from the US National Center for Atmospheric Research Climate Analysis Section website [30]. Only single monthly SOI values are available and are not location-specific. These monthly values were processed to obtain single-week values that were coincident with the weekly malaria data, as described in Buczak et al. [21].

#### Sea surface temperature anomaly

As a complement to SOI values, weekly Sea Surface Temperature Anomaly (SSTA) values were obtained from the NASA Global Change Mastery Directory website [31]. These values are calculated as the area-averaged sea surface temperature anomalies (C) for specified region of the equatorial Pacific Ocean. The Nino 1 + 2 region covers the extreme eastern equatorial Pacific between 0–10S, 90 W–80 W. The Nino-3 region spans the eastern equatorial Pacific between 5 N–5S, 150 W–90 W. The Nino 3.4 region spans the east-central equatorial Pacific between 5 N–5S, 170 W–120 W. The Nino 4 region spans the date line and covers the area 5 N–5S, 160E–150 W. SSTA values are defined as departures from the 1981–2010 base period monthly means. Unlike SOI, SSTA values are typically published for a single week, beginning on Wednesday. To align these values with weekly malaria data (beginning on Sunday), we computed weighted sums as described in Buczak et al. [21, 22]. Both SSTA and SOI can impact regional rainfall patterns over wide areas of the globe, including as far away as Korea [32].

### Techniques

#### Overview

**Fig. 3:**
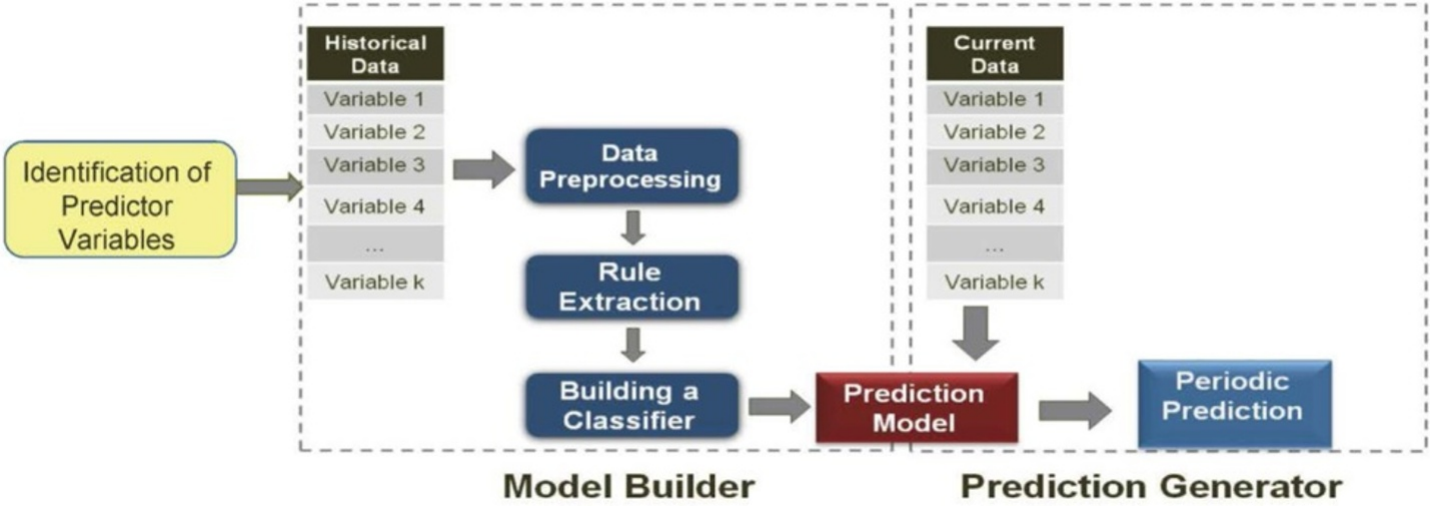
Predicting Infectious Disease Scalable Method (PRISM)

The method performs data mining from a large number of data sources using the steps shown in Fig. 3 [21, 22]:

1. **Identification of the Predictor Variables**: A literature review is performed to identify the environmental and socio-economic variables that are significantly correlated with the given disease, in this case malaria. After the identification of data sources for predictor variables, data are downloaded. The variables used for malaria prediction were described in the earlier Predictor Variables Section. These data are then divided into training, fine-tuning, and testing sets that are used to train and evaluate the prediction model. While the training and fine-tuning sets overlap, the testing set is disjoint from these and is not used at all in the training of the model.
2. **Model Builder**: The Model Builder is the principal part of the method and is where all the data mining elements reside. The data are pre-processed and used to find fuzzy association rules. A subset of these rules that satisfy certain criteria is then selected to create a classifier that becomes the Prediction Model.
  - **Data pre-processing and fuzzification**: The predictor variable data are pre-processed to convert them into the desired spatio-temporal resolution, as described in detail in Buczak et al. [21].
  - **Rule extraction**: Rule extraction from the training data is performed using Fuzzy Association Rule Mining (FARM) [33], where a set of data mining methods that use a fuzzy extension of the Apriori algorithm [34] automatically extract the so-called fuzzy association rules from the data. For the disease prediction application, the rules of interest are called fuzzy class association rules (FCARs), meaning that they have only one consequent: the class. An example of a FCAR extracted by FARM is:
    IF (NK_Funding_Total_Y-3 is ***SMALL***) AND (Malaria_Case_Counts_T-1 is ***LARGE***) AND (Malaria_Case_Counts_T-11 is ***VERY SMALL***) → Malaria_Case_Counts_T + 8 is ***HIGH***, confidence = 0.899, support = 0.0011, lift = 20.83
    The above rule states that if total funding obtained by DPRK for malaria eradication three years ago was ***SMALL***, and Malaria Case Counts one week ago (T-1) were ***LARGE***, and Malaria Case Counts eleven weeks ago (T-11) were ***VERY SMALL***, then the Malaria Case Counts will be ***HIGH*** 8 weeks from now (T + 8). The terms confidence, support, and lift are metrics used in the rule selection to be described next.
  - **Rule selection**: The FARM method typically extracts thousands of rules, but only a subset of these is used to build a classifier that becomes the final prediction model. An automatic method is used to choose a small subset of rules that minimizes the misclassification error on the fine-tuning data set. The rule choices are based on selection criteria using the three most important metrics for fuzzy association rules: confidence, lift, and support. Confidence is the conditional probability that, if the antecedents are true, then the consequent is true. A rule with confidence of 1 is always true. Support is a measure of how general a given rule is and can be considered to be the probability of occurrence of records with given antecedents and consequent in a particular data set. A support of 0.01 means that a given rule describes 1 % of a particular data set. Lift represents the extent to which the antecedents and the consequents are not independent. The higher the lift, the more dependent the variables are. A thorough description of the rule metrics and associated equations can be found in [34, 21].
3. **Prediction Generator**: The final classifiers using rules selected from the previous step become the models that generate predictions. These models are evaluated using measures described in the section called Performance Metrics. A final prediction model is selected based on these metrics and the desires of the end user. In the case of ROK, the users requested predictions for certain case count ranges (LOW, MEDIUM, and HIGH, to be subsequently described) and for 7–8 weeks in advance for certain regions near the DMZ.

#### Rule generation improvements

Buczak et al. [21] used a fuzzified version of the Apriori algorithm [34] to mine the Fuzzy Class Association Rules (FCARs) that were subsequently used to build the classifiers. This algorithm works well in the classical association rule mining setting where the data set is typically sparse, the support threshold is set sufficiently high to ensure that there are only a manageable number of frequent sets, and the goal is to mine all of the frequent sets in order to discover interesting association rules. However, when attempting to predict relatively rare disease outbreaks, we are also very interested in rare (infrequent) but strong (high confidence) association rules because these are vital in building a sufficiently sensitive classifier. This requires the support thresholds to be set very low, which causes a combinatorial explosion in the number of frequent sets even for relatively small data sets. Also the data sets tend to be much denser than transaction databases, which increases further the number of frequent sets.

One of the underlying assumptions of the Apriori algorithm is that parsing the data set is more expensive than parsing the rule set. This is why it builds size *k* frequent sets by combining size *k*-1 frequent sets that share *k*-2 items. Doing so minimizes the number of passes through the data set because of the Apriori principle, but requires the rule set to be parsed multiple times. When the rule set grows significantly larger than the data set, then this approach is no longer effective. In order to use the Apriori algorithm in the disease prediction setting, Buczak et al. [21] had to set a relatively low upper bound on the size of the frequent sets due to computer memory constraints. Although the use of the low support thresholds allowed for the mining to discover some interesting rare rules and build an effective classifier, the low upper bound was a significant limitation and hindered the performance of the model.

Therefore, an improved algorithm was developed that is better suited to mine for rare, high confidence FCARs in dense data sets and can scale up to very large rule sets. In this algorithm, FCARs are mined directly and the support and confidence of a large number of FCARs are computed on each pass through the database. The algorithm essentially performs a breadth-first search through the trees of all possible FCARs, pruning nodes that either fall below the minimum support threshold or achieve a confidence within a user-defined threshold of 1. In the former case, all children of the node must also fall below the support threshold and in the latter case all children of the node would later be removed in the subsequent pruning step. At each step, all size *k* + 1 children of a block of size *k* rules are evaluated on each pass through the database and this processing is parallelized. When certain levels of the tree are reached (either pre-defined by the user or determined at run-time), the current search splits and a breadth-first search is initiated on each live node at that level. This feature is not only useful in reducing the memory footprint of the process but also makes it well suited to distributing out to a cloud of computer nodes if needed. At the end of each search, a confidence-based pruning is performed on the set of rules, which drastically reduces the number of rules that need to be passed along to the classifier generation algorithm. Any rule whose confidence is less than or within a user-defined threshold of the confidence of a parent rule is pruned.

#### Classifier improvements

Buczak et al. [21] described a slightly modified version of the method of Liu et al. [35] to build a classifier from the FCARs. In Buczak et al. [22], some improvements to this implementation were presented. For the present study, additional enhancements to the classifier-building algorithm were made.

The first enhancement was to develop a new classifier generation algorithm tailored to the Weighted Voting Classifier described in Buczak et al. [22]. The new algorithm takes as input the set of fuzzy association rules that have been mined from the training data. It separates these by class and then ranks the class lists individually. The original algorithm would often generate classifiers with a disproportionate number of rules from one of the classes. This is not a problem for a decision list classifier since only one rule is used to make the classification, but it is easy to see why this poses a potential problem for a voting classifier. For the new generation algorithm, we wanted to ensure that there would be a more balanced set of rules. Therefore, at each step the algorithm adds a rule from each class that has not met its stopping condition. The criteria for adding a rule to the classifier is that it be the highest ranked rule remaining in its class that improves the performance of the classifier on the training data. In other words, if adding its votes on the data to the current rules’ votes decreases the misclassification score of the classifier, then it is added to the classifier. The misclassification score is calculated by multiplying the number of times a class was misclassified by the misclassification weight for that class and then summing across the classes. The algorithm stops when all the data points have been classified, there are no remaining rules that improve the classifier, or the stopping condition for all classes has been met. The stopping condition was added to help limit the overfitting of the training data [36]. It is an optional user-defined parameter that defines for each class the proportion of data points in the training set that should be covered. Once the defined proportion is covered for a particular class, no more rules are added to the classifier for that class.

A second enhancement was to add an additional rule ranking method. The rules are ranked from best to worst prior to being passed to the classifier generation algorithms. The original method ranked first by confidence, then by support, then by lift, and lastly by number of antecedents. This method works fairly well; however, it always gives confidence the top priority. This is fine if the confidence value is accurate, but since the rules are mined from data that are very noisy, the confidence values can be unreliable, especially for rules with very low support. For example, we would intuitively trust a rule with confidence = 0.999 and support = 0.1 much more than a rule with confidence = 1 and support = 0.001; however, the current ranking method would always rank the second rule higher. Therefore, we thought that at least in some cases performance could be improved by using a ranking method that relied on a metric that balances confidence and support. We tried a few methods and determined that the pessimistic error rate developed by Quinlan [37] was best suited for this task.

### Performance metrics

For a two-class problem, four metrics were used to assess the accuracy of the prediction [21, 22]:

- Positive Predictive Value (PPV): PPV = TP/(TP + FP) which is the proportion of positive predictions that are outbreaks;
- Negative Predictive Value (NPV): NPV = TN/(TN + FN) which is the proportion of negative predictions that are non-outbreaks;
- Sensitivity: Sensitivity = TP/(TP + FN) which is the proportion of correctly predicted outbreaks (also called Probability of Detection);
- Specificity: Specificity = TN/(TN + FP) which is the proportion of correctly predicted non-outbreaks; 1- Specificity is the False Alarm Rate;

where TP, TN, FP, and FN represent, respectively, True Positive, True Negative, False Positive, and False Negative.

These measures of accuracy may then be used to select a prediction model that best meets the needs of the user. For example, a high PPV indicates that, when the model predicts high incidence rate, it is likely that a high incidence will actually occur. A high PPV may be desirable when disease prevention and mitigation resources are limited. A high Sensitivity indicates that the model predicts a high percentage of the outbreaks that actually occur. Therefore, the F-score [38] is used as a measure that considers both PPV and Sensitivity:

1 $$ {F}_{\beta }=\left(1+{\beta}^2\right)\frac{PPV\ast Sensitivity}{\beta^2\ast PPV+ Sensitivity} $$

By varying the value of *β*, the resulting F-score will reflect the relative importance given to PPV and Sensitivity. Therefore, F0.5 (PPV more important) and F3 (Sensitivity more important) values were calculated to reflect the performance of the models.

For a multi-class classification problem (with number of classes larger than two) the Sensitivity and PPV for each class are widely used [39]:

2 $$ Sensitivit{y}_j=\frac{T{P}_j}{T{P}_j+F{N}_j} $$

3 $$ PP{V}_j=\frac{T{P}_j}{T{P}_j+F{P}_j} $$

where TP_j_, FN_j_, FP_j_ correspond to True Positive, False Negative and False Positive for class *j*, respectively. The per-class F-score can be computed using equation (1), in which the class Sensitivity and PPV from equations (2) and (3) are used.

## Results and discussion

### Incidence prediction vs. case count prediction

**Fig. 4:**
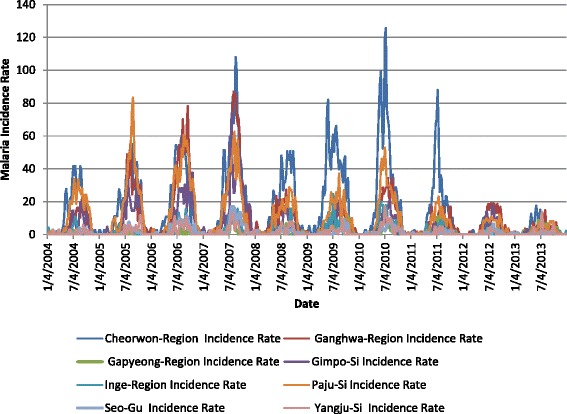
Malaria incidence rate for eight example regions

In previous work [21, 22], the disease incidence rate was used to classify periods of time into ***HIGH*** or ***LOW*** incidence. The incidence rate normalizes the data by the using the region population and is defined as:

4 $$ \mathrm{Incidence}\ \mathrm{Rate} = \upalpha *\left(\mathrm{new}\ \mathrm{reported}\ \mathrm{case}\ \mathrm{counts}\right)/\mathrm{population} $$

where *α* is some constant scaling factor. The high and low classes were determined by selecting a threshold to divide the data into the two classes. This incidence rate threshold was calculated using the training data for all regions and was computed as

5 $$ T = \mu + \beta \sigma $$

where *μ* was the mean, *σ* was the standard deviation and *β* was some constant. Figure 4 plots the malaria incidence rates for eight Korean regions. Notice that the dark blue peaks in the 5th through 8th years are much higher than the others and that this characteristic increased both the mean and standard deviation used for computing the threshold. Although the resulting incidence rate threshold worked for providing two-class training data, the outlier peaks in years 5 through 8 skewed the threshold computation so that it was too large to provide enough ***HIGH*** class samples for the validation and testing years of data.

**Fig. 5:**
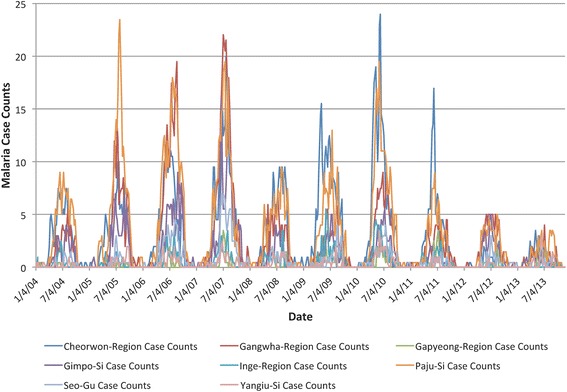
Malaria case counts for eight example regions

Because of this problem using incidence rate, case counts were examined instead as a possible data source for determining the classes. Figure 5 plots the case counts for the same eight Korean regions. Notice that for the 5th through 8th years, the dark blue case count values do not overwhelm the other region counts. Therefore, using case counts for computing the threshold defined above and applying the threshold to the case counts data provided enough high values for training, validation, and testing. Therefore, the ***HIGH*** incidence classifier was developed, trained, and tested using eight regions and the threshold based on case counts data.

### FARM results

In the ROK malaria dataset, there is a huge variation in the yearly case counts and most years are at one extreme or the other (note the variations from year to year in Fig. 2). In addition, the most recent years (which are the most natural ones to use for testing) had the lowest counts. It was immediately clear that predicting just two classes (i.e., ***LOW***, ***HIGH***) as in our previous work [21, 22] would result in the data having only lows in 2013. This resulted in a prediction accuracy of 100 % (all were LOW), but this obviously has limited usefulness. Because of the nature of this dataset, better overall model performance could be achieved when different training and testing sets were used for training a classifier for medium weekly case numbers than those testing sets used for training classifiers for high weekly case numbers. Thus, two separate binary classifiers (one for ***HIGH*** and one for ***MEDIUM***) were trained and their output was combined to create the final classifier to provide three classes: ***LOW***, ***MEDIUM***, and ***HIGH***. In addition, the public health users in ROK expressed a preference for three classes, with HIGH corresponding to an outbreak.

As mentioned above, the number of malaria cases was relatively low in 2012 and 2013. For example, the year 2013 had no periods with a high number of cases in any of the regions. In order to effectively train and test a model for high weekly case numbers, a year with a relatively high number of cases was included in both the fine-tuning and testing sets. The training data for the high classifier included 8 regions and spanned January 2004–December 2006 and January 2008-December 2012. The fine-tuning data included January 2006–December 2006 and January 2012–December 2012 data for the same 8 regions. The testing data spanned January 2007–December 2007 and January 2013–December 2013 for these 8 regions. The results reported in the second row of Table 1 are based only on the performance of the models in predicting the 2007 and 2013 weekly case numbers data that were not used for model development.

**Table 1:** Performance of two-class classifiers on test data for predictions made 7–8 weeks ahead. Confidence intervals computed for α = 0.05

Data set	PPV	NPV	Sensitivity	Specificity	F0.5	F3
2007 & 2013 (HIGH Classifier)	0.842	0.981	0.681	0.992	0.804	0.694
Lower Conf Bound	0.696	0.969	0.538	0.983	0.657	0.551
Upper Conf Bound	0.926	0.989	0.796	0.996	0.896	0.807
2007 & 2013 (MEDIUM Classifier)	0.791	0.962	0.374	0.994	0.647	0.395
Lower Conf Bound	0.726	0.958	0.327	0.992	0.584	0.346
Upper Conf Bound	0.844	0.967	0.423	0.996	0.704	0.446

The training data for the medium classifier included 64 regions and spanned January 2004–December 2006 and January 2008-December 2012. The fine-tuning data included January 2006–December 2006 and January 2012–December 2012 data for the same 64 regions. The testing data spanned January 2007–December 2007 and January 2013–December 2013 for these 64 regions. The results reported in the third row of Table 1 are based only on the performance of the models in predicting the 2007 and 2013 weekly case numbers data that were not used for model development.

The two binary classifiers described above were then combined into a multi-class classifier by applying the high classifier first, followed by the medium classifier only when the outcome of the high classifier was negative. The Table 2 shows the simple decision logic.

**Table 2:** Classifier fusion

High classifier outcome	Med classifier outcome	Final class
HIGH	MEDIUM	HIGH
HIGH	LOW	HIGH
LOW	MEDIUM	MEDIUM
LOW	LOW	LOW

Table 3 provides the per-class results for the combined classifier that are based only on the performance of this multi-class model for 64 regions on the 2007 and 2013 weekly case number data that was not used in the development of either classifier. Per-class results are obtained by applying equations (2) and (3) to each class. Table 4 shows F0.5 and F3 for each class obtained using equation (1). It is relatively easy to obtain good results for the ***LOW*** class for which there are a lot of exemplars. It is the most difficult to obtain good results for the ***HIGH*** class and therefore this is the class on which we are concentrating the most.

**Table 3:** Sensitivity and PPV for the FARM method for predictions made 7–8 weeks ahead. Confidence intervals computed for α = 0.05

	Sensitivity	Sensitivity	Sensitivity	PPV	PPV	PPV
	LOW	MEDIUM	HIGH	LOW	MEDIUM	HIGH
Value	0.993	0.275	0.681	0.963	0.637	0.842
Lower Conf Bound	0.992	0.230	0.538	0.958	0.556	0.696
Upper Conf Bound	0.995	0.325	0.796	0.967	0.711	0.926

**Table 4:** F values for the FARM method for predictions made 7–8 weeks ahead. Confidence intervals computed for α = 0.05

	F0.5	F3	F0.5	F3	F0.5	F3
	LOW	LOW	MEDIUM	MEDIUM	HIGH	HIGH
Value	0.969	0.991	0.504	0.292	0.804	0.694
Lower Conf Bound	0.965	0.988	0.433	0.244	0.657	0.550
Upper Conf Bound	0.972	0.992	0.575	0.344	0.897	0.807

**Fig. 6:**
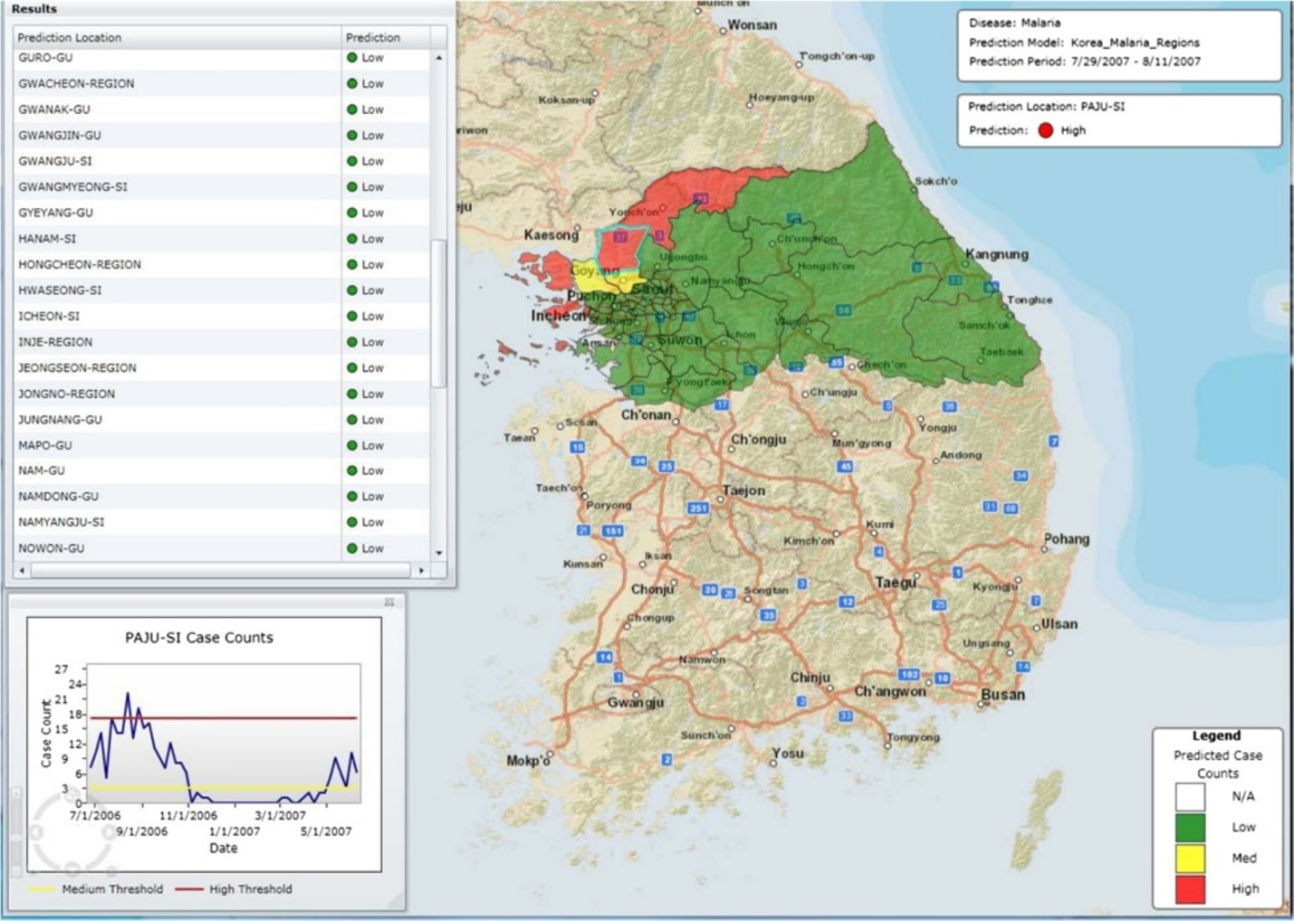
Seven to eight weeks ahead prediction for northern regions of South Korea for the period 7/29/2007-8/11/2007

**Fig. 7:**
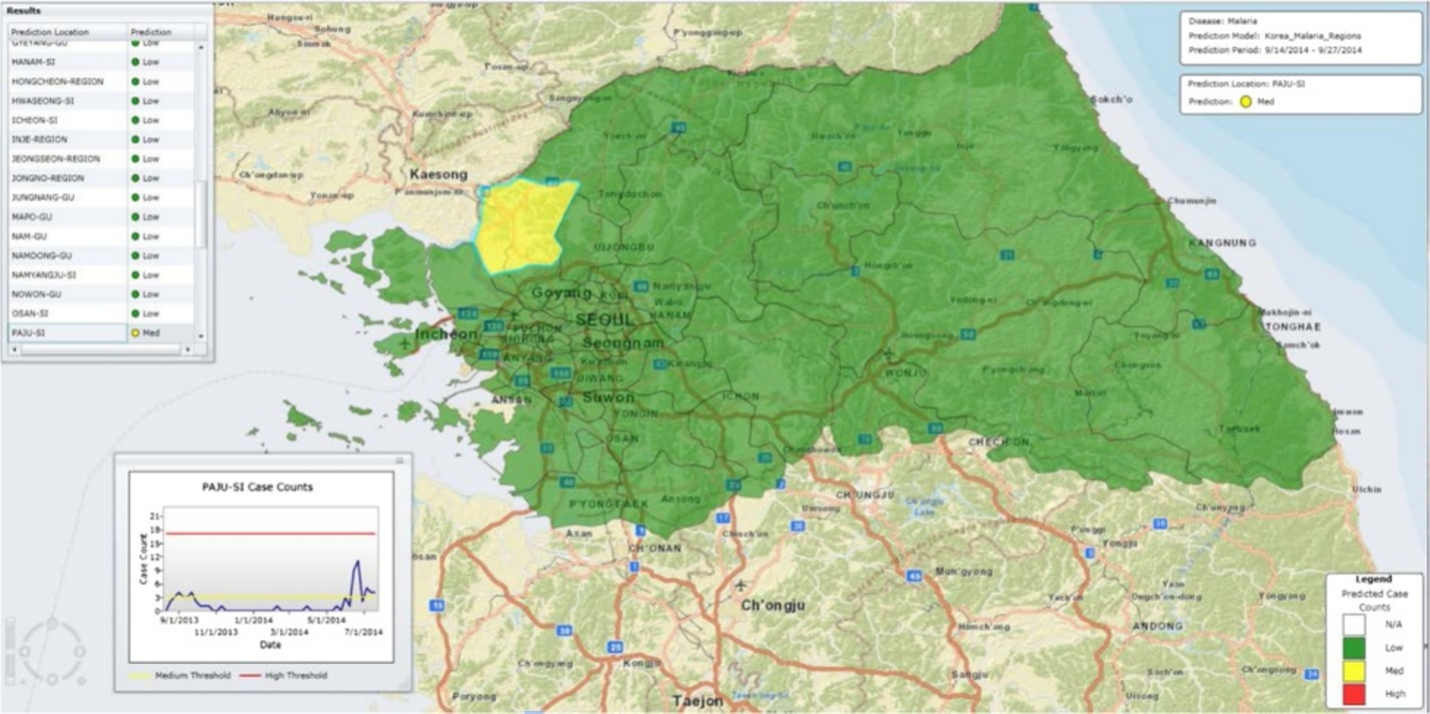
Seven to eight weeks ahead prediction for northern regions of South Korea for the period 9/14/2014-9/27/2014

Figures 6 and 7 show 7–8 weeks ahead predictions for the northern regions of South Korea for two different two-week intervals. In Fig. 6 some regions are predicted as ***HIGH***, some ***MEDIUM***, and some ***LOW***. On Fig. 7 one region is predicted as ***MEDIUM*** but the others are predicted as ***LOW***. Current predictions can be found on the Predicting Infectious Diseases Scalable Method (PRISM) website [40].

### Other methods’ results

In order to compare the performance of the FARM-based method with some well-known classifiers, Decision Tree, Random Forest, and Support Vector Machine (SVM) classifiers were trained. In addition, a Holt-Winters exponential smoothing model was developed.

A Decision Tree is a tree-like structure with leaves representing classifications and branches representing the combinations of feature values that lead to those classifications. A data point is classified by testing its feature (attribute) values against the nodes of the decision tree. The best-known methods for automatically building decision trees are the ID3 algorithm [41] and the C4.5 algorithm [42]. Both algorithms build Decision Trees from a set of training data using the concept of information entropy. When building a Decision Tree, at each node of the tree, C4.5 chooses the attribute of the data that most effectively splits its set of examples into subsets. The splitting criterion is the normalized information gain (difference in entropy). The attribute with the highest normalized information gain is chosen to make the decision.

The next classifier used for comparison is a Random Forest [43]. The Random Forest classifier is a machine learning method that combines decision trees with ensemble learning. The forest is composed of many Decision Trees that use randomly picked data attributes as their input. The forest generation process constructs a collection of trees with controlled error variance. The resulting prediction can be decided by a selection scheme, such as majority voting. For instance, the Weka [44] implementation averages the class probability estimates from each tree to make a prediction.

The third classifier used is an SVM. The SVM finds a separating hyperplane in the feature space between two classes in such a way that the distance between the hyperplane and the closest data points of each class is maximized. The approach is based on a minimized classification risk [45] rather than an optimal classification. SVMs are well known for their generalization ability and are particularly useful when the number of features, *m*, is high and the number of data points, *n*, is low (*m* > > *n*). Various types of dividing hyperplanes can be realized by applying a kernel, such as linear, polynomial, Gaussian Radial Basis Function (RBF), or hyperbolic tangent. In this work, the RBF kernel was used. SVMs are binary classifiers and multi-class classification is realized by developing an SVM for each pair of classes.

In this work, Weka [44] implementations were used for all three classifiers. A two-dimensional grid search is performed to optimize the most important classifier parameters. Default values from the Weka toolbox for the respective classifiers are used for the remaining parameters. Each trained classifier is evaluated on the validation data to determine the best model parameters and the model with those best parameters is used for testing on the test data set. For the Decision Tree, the minimum number of parameters per leaf and the number of folds for reduced error pruning are optimized. For the Random Forest, the number of trees and the number of random features per tree are optimized. For the SVM model, the non-separable cost parameter and the Radial Basis Function gamma parameter are optimized.

Another method used for malaria prediction is exponential smoothing with seasonality introduced using the Holt-Winters procedure [46]. The Holt-Winters seasonal method comprises the forecast equation and three smoothing equations, with smoothing parameters α, β, and γ. The weekly malaria counts were transformed via the logarithmic transformation z_t_ = log (y_t_ + 1) (where y_t_ is the count and z_t_ is the transformed count) on which the exponential smoothing was performed. A separate exponential smoothing model using additive Holt-Winters was needed for every region in the data set (64 regions). For each region, all the consecutive data from the preceding years present in the training set were used for model development. Thus, for predicting 2007, data from a given region from the period 2004–2006 were used; for predicting 2013, data from 2008–2012 were used. Covariates such as rainfall and temperature were not used because Briet et al. [20] established that they don’t consistently improve the results of Holt-Winters prediction, and sometimes even make the prediction worse.

The values of α, β, and γ were optimized on the training data set together for all the regions in such a way as to minimize the mean absolute relative error (MARE). The outputs of exponential smoothing models are continuous numbers, not categories as in the machine learning models above. In order to be able to compare these results with the three class results of FARM and other machine learning methods, the results were binned into the categories ***LOW***, ***MEDIUM***, and ***HIGH*** using the thresholds used for FARM: 3 and 17. Because the data were log transformed, we are actually using log (3 + 1) and log (17 + 1) as thresholds for MEDIUM and HIGH, respectively. In case of FARM, Random Forest, Decision Tree, and SVM, one classifier is trained and used for all 64 regions. In case of Holt-Winters exponential smoothing, a separate model was needed for every region (otherwise the results would have been much worse than presented here). While the other models used multiple temporal, as well as spatial, variables, only case counts were used in case of prediction using Holt-Winters exponential smoothing. Because this model is very different from the machine learning models, its comparison with machine learning models is not exactly straightforward. For the Holt-Winters method, the predictions for each region and year are generated by separate models (separate data for only a given region and appropriate time period used) and thus the final prediction metrics (PPV and Sensitivity) are obtained by taking the mean of these metrics from the individual models.

**Fig. 8:**
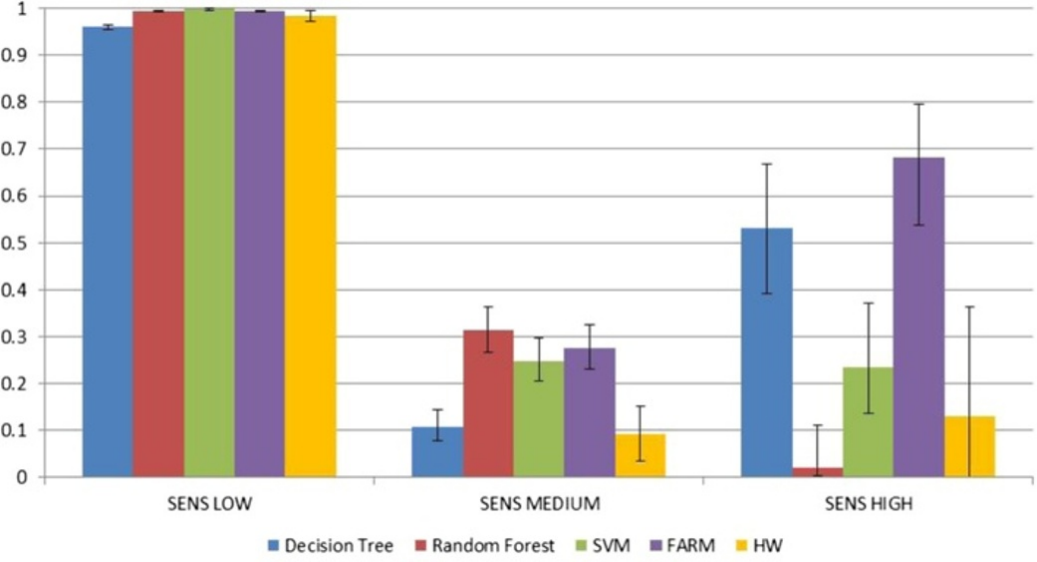
Sensitivity of *LOW*, *MEDIUM* and *HIGH* classes. Confidence intervals shown for α = 0.05

**Fig. 9:**
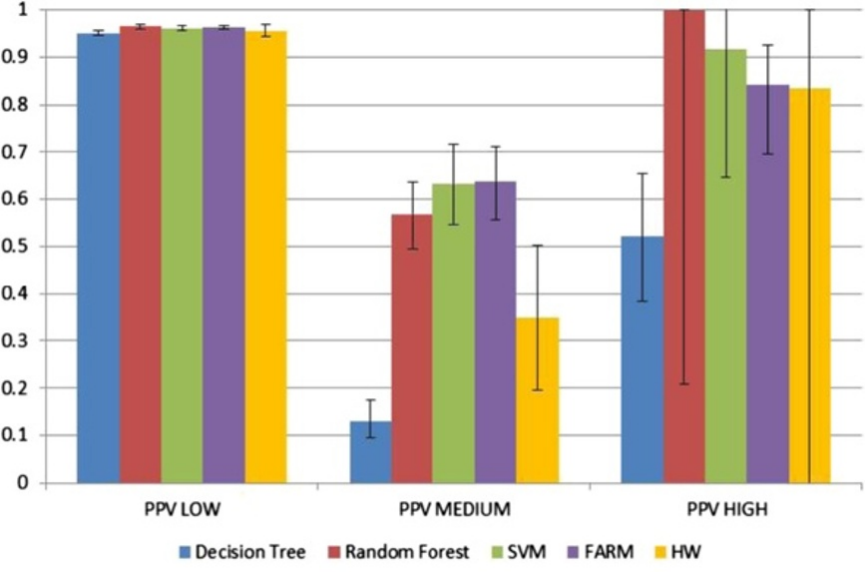
PPV of *LOW*, *MEDIUM*, and *HIGH* classes. Confidence intervals shown for α = 0.05

Figure 8 shows per-class Sensitivity for classes ***LOW***, ***MEDIUM*** and ***HIGH***. Figure 9 shows the PPV for the same classes. The confidence intervals for α = 0.05 were computed using the Wilson method [47] for all the prediction models, except for Holt-Winters where they are confidence intervals on the estimate of the population mean using the t-distribution. Both methods remain valid even for small sample sizes. However, as can be seen in the case of ***HIGH*** PPV for both the Random Forest and Holt-Winters, the confidence intervals become very large for very small sample sizes.

In the entire test data set, the vast majority of exemplars are from the ***LOW*** class because it is easiest to get good Sensitivity and PPV for that class due to high prevalence of LOWs in the data. As shown in Figs. 8 and 9, all five methods get good results for the ***LOW*** class. For the ***MEDIUM*** class, the best Sensitivity was obtained by Random Forest, second best by FARM, and third best by SVM (the results of those three methods are very close and their confidence intervals are very close as well). The best PPV was obtained by FARM, with SVM being only slightly lower (with FARM confidence intervals being slightly narrower than those of SVM).

The prediction of the ***HIGH*** class is considered to be the most important because this is how we define an outbreak and is based on public health officials indicating that they would be most likely to take action when ***HIGH*** is predicted. For the ***HIGH*** class, the best Sensitivity results were obtained by FARM, and the next best by Decision Tree. In contrast, the worst Sensitivity results were obtained by Random Forest (0.043), which predicted only two out of 47 ***HIGH*** cases. For the ***HIGH*** class, Random Forest performed the best in terms of PPV, with SVM being the second best, and FARM being the third best. While Holt-Winters had only slightly worse results than FARM, its confidence interval was very large due to the fact that only 2 of the 128 Holt-Winters models predicted any ***HIGH***s. Therefore, the final Holt-Winters PPV was the mean of only 2 values, necessitating the use of a large t-value (drawn from the t-distribution with only 1° of freedom, which has very fat tails due to the variability associated with using such a small sample size). This large t-value, combined with a rather large standard deviation, resulted in a confidence interval covering the entire range of values.

**Fig. 10:**
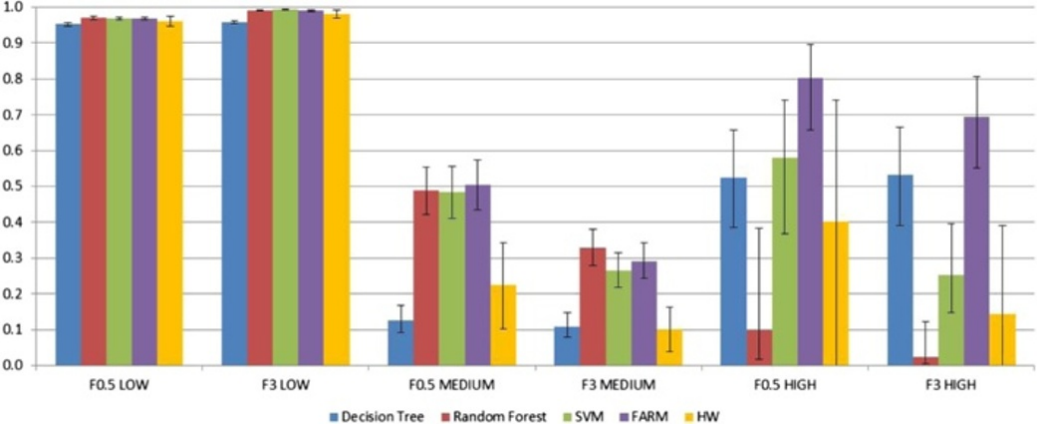
F measures for *LOW*, *MEDIUM*, and *HIGH* classes. Confidence intervals shown for α = 0.05

It is important to remember that looking at Sensitivity and PPV separately can be misleading because both values need to be high enough for the classifier to be useful in practice. This is why F-scores are used. For example, Random Forest for the ***HIGH*** class has a PPV of 1, but its Sensitivity is a dismal 0.043. Many of the methods had a high PPV and low Sensitivity or vice versa. The method that consistently gets high values of both PPV and Sensitivity is FARM. Note in Fig. 10 that the F-scores are consistently large for FARM, especially for the ***HIGH*** class. As mentioned previously, the overall best results are chosen based on F0.5 and F3 metrics that combine PPV and Sensitivity (see Fig. 10). Because the metrics for the ***LOW*** class are very close to 1 for all methods as explained earlier, they are not taken into consideration. Three metrics (F0.5 ***MEDIUM***, F0.5 ***HIGH***, and F3 ***HIGH***) are the highest for FARM, with the metrics for ***HIGH*** being better than those for ***MEDIUM***. F3 ***MEDIUM*** is the highest for Random Forest. The confidence intervals on the F-scores were computed using a combination of the confidence intervals on the PPV and Sensitivity. Large confidence intervals on the F-scores in the case of Random Forest and Holt-Winters were caused by correspondingly large confidence intervals on the PPVs or Sensitivities as detailed above.

There is a significant difference between the performance of the ***MEDIUM*** classifier in Table 1 and the performance of the fused classifier on the ***MEDIUM*** class in Table 3. This is because the composition of the ***MEDIUM*** class is different in these two cases. In the training set for the binary ***MEDIUM*** classifier (Table 1), all points above the ***MEDIUM*** threshold are in the ***MEDIUM*** class, but for the ternary fused classifier (Table 3), only those between the ***MEDIUM*** and ***HIGH*** thresholds are in the MEDIUM class. The ***MEDIUM*** classifier naturally has better performance on the points farther from the decision boundary than on those closer to it. This lower performance on the points close to the boundary is what is reflected in the final performance metrics of the fused classifier. In future work, we hope to be able to address this discrepancy by altering the training method of the ***MEDIUM*** classifier.

## Conclusions

This paper describes the extension of the method previously developed for the creation of dengue prediction models [21, 22] to a different disease, malaria. The extension involved algorithmic changes to the classifier-building algorithm that creates the new model. While the model creation technique is similar (except for these algorithmic changes) for all diseases, the final malaria model is not the same as the earlier dengue models. The set of variables identified for the malaria model were based on published malaria literature and not dengue literature. In addition, new variables specific to ROK predictions were used, including DPRK malaria yearly data, DPRK mosquito net data, and financial data for DPRK malaria control, as well as distances from ROK regions to DMZ. One of the challenges of this work is that the malaria cases in ROK were significantly decreasing in the last two years, resulting in not enough samples of ***HIGH*** cases. However, the development of new classifier building methods and data fusion from two classifiers enabled the creation of a prediction model for malaria in the northern regions of ROK, which are the areas of ROK that see most of the malaria cases in the country. The model creation technique described herein results in a new model capable of taking into account complicated relationships among predictor variables. The result is a model that successfully predicts malaria cases 7–8 weeks in advance using performance metrics that do not involve data used in model development and therefore provide for more conservative and less biased estimates of model performance for the user (Tables 3 and 4).

The data mining techniques used to create prediction models are general in the sense that they can use any data. The method automatically selects association rules that meet pre-defined criteria. These pre-defined criteria are based upon user needs (e.g., low false positives) and select the most important rules that are used in the final disease prediction model, which is objective and reproducible. The data are used according to the dates that they are actually available to the user, so there is no need to assume all data are immediately available, which is often not the case operationally. Provided that sufficient data of reasonable quality are available, using this method to create new models to predict high/medium/low disease incidence for other mosquito-borne diseases is expected to provide similar performance.

## Abbreviations

CDC: US Centers for Disease Control and Prevention
DMZ: Demilitarized Zone (between North and South Korea)
DPRK: Democratic People’s Republic of Korea (North Korea)
ENSO: El Nino Southern Oscillation
EVI: Enhanced Vegetation Index
FARM: Fuzzy Association Rule Mining
FCAR: Fuzzy Class Association Rule
FN: False Negative
FP: False Positive
GIS: Geographic Information System
MARE: Mean Absolute Relative Error
MODIS: Moderate Resolution Imaging Spectrometer
NASA: US National Aeronautics and Space Administration
NDVI: Normalized Difference Vegetation Index
NOAA: US National Oceanic and Atmospheric Administration
NPV: Negative Predictive Value
*P.*: *Plasmodium*
PPV: Positive Predictive Value
RBF: Gaussian Radial Basis Function
ROK: Republic of Korea (South Korea)
SOI: Southern Oscillation Index
SST: Sea Surface Temperature
SSTA: Sea Surface Temperature Anomaly
SVN: Support Vector Machine
TN: True Negative
TP: True Positive
TRMM: Tropical Rainfall Measuring Mission
USGS: US Geological Survey
WHO: World Health Organization
